# Age-related differences in ocular biometry in adult Korean population

**DOI:** 10.1186/s12886-016-0328-8

**Published:** 2016-08-22

**Authors:** Jung-Hoon Kim, Moosang Kim, Seung-Jun Lee, Sang Beom Han, Young Tae Kong, Hee Kyung Yang, Joon Young Hyon

**Affiliations:** 1 Department of Ophthalmology, Kangwon National University Hospital, Kangwon National University Graduate School of Medicine, 156 Baengnyeong-ro, Chuncheon, Kangwon 200-722 Korea; 2 Kong Eye Center, Seoul, Korea; 3 Department of Ophthalmology, Seoul National University College of Medicine, Seoul, Korea

**Keywords:** Anterior chamber depth, Age, Axial length, Keratometry, Ocular biometry

## Abstract

**Background:**

To evaluate the relationship between age, axial length (AL), anterior chamber depth (ACD) and corneal steepness (K) in patients with cataract.

**Methods:**

In this retrospective study, medical records of 800 patients (800 eyes) who were diagnosed with cataract and received preoperative ophthalmologic examination were reviewed. Data including age, gender and ocular biometric data including AL, ACD and K were collected and analyzed using univariate and multivariate analyses.

**Results:**

Univariate analysis showed increased age has significant correlation with shorter AL (*P* < 0.001), shallower ACD (*P* < 0.001) and steeper K (*P* < 0.001). *K* value has a negative correlation with AL (*P* < 0.001). In multivariate analysis, increasing age has a significant association with shorter AL (*P* < 0.001) and ACD (*P* < 0.001), although the association between age and K was not significant (*P* = 0.398). Negative correlation between AL and K remained significant in multivariate analysis (*P* < 0.001).

**Conclusion:**

In patients with cataract, older age had significant association with shorter AL and ACD. AL and K had negative correlation.

## Background

Structures in a human eye change with advancing age [1]. Half of the postnatal eye growth occurs within the first 12–16 months after birth, and most ocular structures continue developing until the age of 13–14 years [2, 3] Axial length (AL) increases rapidly till 3 years of age, after which the growth slows down to 0.16 mm/year for age–9 years, 0.08 mm/year for age 9–12 years and 0.02 mm/year for age 11–14 years [3, 4]. After age 15, ocular parameters including AL, corneal diameter, and corneal curvature remain stationary, although AL elongation sometimes continues at age of 13–18 years [3, 5].

However, the changes of ocular parameters in adults, especially in elderly subjects, are not clearly understood yet. Although several studies have been conducted to investigate the changes in ocular biometry with aging, there were controversies between the results of the studies [6–17]. Thus, the age-related variation of ocular biometric data including AL, anterior chamber depth (ACD) and corneal curvature in adult was not fully elucidated.

In the present study, we evaluated the relations between age, AL, ACD and corneal steepness in adult subjects who were diagnosed with cataract and received preoperative examination for cataract surgery.

## Methods

### Study participants

This is a retrospective study that included 800 eyes of 800 patients who underwent cataract surgery at Kangwon National University Hospital and Kong Eye Center from March 2013 to May 2014 and received preoperative ophthalmologic examination. Exclusion criteria included 1) media opacity including posterior or anterior capsular cataract, mature cataract or vitreous opacity in which measurement using an optical biometer (IOL Master®; Carl Zeiss Meditec, Oberkochen, Germany) with a signal to noise ratio of 2.0 or greater could not be acquired 2) corneal diseases that can affect corneal curvature, such as, pterygium, corneal degeneration or dystrophy 3) eyes with AL < 22 mm or > 27 mm 4) myopia < −6 diopters or hyperopia > +4 diopters 4) intraocular diseases that can affect AL and ACD or make accurate measurement of AL difficult, such as, epiretinal membrane, macular edema or degeneration, glaucoma, or intraocular tumor 5) history of ocular surgery or trauma 6) age of younger than 20 years.

Medical records including age and gender were collected. Ocular biometric data including AL, ACD, and keratometry (K) values were measured before cataract operation using an optical biometer (IOL Master®; Carl Zeiss Meditec, Oberkochen, Germany). AL was measured as the distance from the anterior corneal vertex to the retinal pigment epithelium (RPE) along fixation, automatically adjusted for the distance between the inner limiting membrane and RPE [17]. ACD was measured as the distance from the anterior corneal vertex to the anterior lens surface by image analysis of an optical section. The *K* value was measured along the two meridians, the greatest and least radii of curvature (K1, K2), and the average of the two values was taken as the *K* value. The measurements were performed by a masked technician.

### Statistical analysis

The associations between age, AL, ACD and K were analyzed using SPSS for Windows (version 18.0, SPSS Inc., Chicago, IL, USA). The correlation between age and each parameter were analyzed using Pearson correlation analysis. Differences in ocular parameters according to age and AL were evaluated using one-way analysis of variance (ANOVA). Post hoc pair-wise comparisons adjusted by the Bonferroni-Dunn method were also performed among various age and AL groups. Multivariate analysis using linear regression analysis was performed to determine the correlation between each parameter. *P* values < 0.05 were considered statistically significant.

## Results

The study patients consisted of 406 men (50.8 %) and 394 women (49.3 %), and the mean age was 67.4 ± 12.0 years (mean ± SD; range, 20–94 years). The average AL, ACD and K value were 23.59 ± 0.89 mm, 3.01 ± 0.47 mm, and 44.16 ± 1.40 D, respectively.

**Fig. 1:**
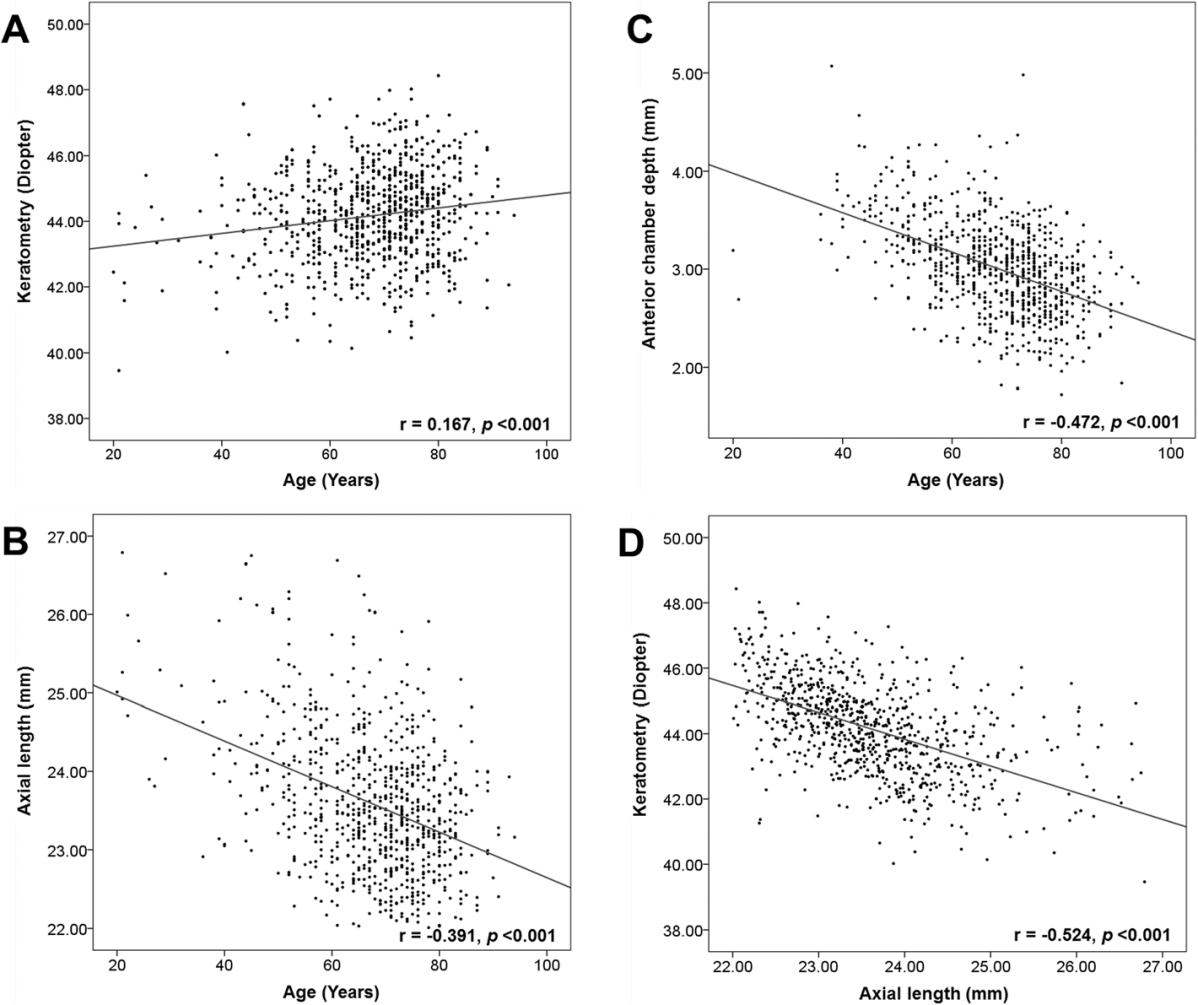
Correlations between age and ocular parameters. Pearson correlation analysis showed significant correlation between advancing age and steeper keratometry value (**a**), shorter axial length (**b**), and shallower anterior chamber (**c**). Axial length had a negative correlation with keratometry value (**d**)

Univariate analysis using Pearson correlation analysis demonstrated that older age had significant association with steeper K (*r* = 0.167, *P* < 0.001), shorter AL (*r* = −0.391, *P* < 0.001) and shallower ACD (*r* = −0.472, *P* < 0.001). AL and K had a negative correlation (−0.524, *P* < 0.001) (Fig. 1). Regarding the gender, no significant difference was found in AL (23.59 ± 0.90 mm (men) vs. 23.60 ± 0.89 mm (women), *P* = 0.916), ACD (3.00 ± 0.45 mm (men) vs. 3.02 ± 0.49 mm (women), *P* = 0.620) or K (44.11 ± 1.40 D (men) vs. 44.21 ± 1.35 D (women), *P* = 0.300) between men and women. There was no significant difference in age (67.9 ± 12.0 years 9 (men) vs. 66.8 ± 12.1 years (women)) according to gender.

One-way ANOVA showed that there are significant differences in AL, ACD and K between various age groups (F = 31.84, 45.01 and 4.59 and for AL, ACD and K, respectively; *P* < 0.001 for all parameters) (Table 1). Post hoc pair-wise comparisons adjusted by the Bonferroni-Dunn method demonstrated that AL was significantly different in every comparison except between age groups of ≤ 39 years and 40 to 49 years and between groups of 70–79 years and ≥ 80 years. ACD was significantly different in every comparison except between age groups of ≤ 39 years and 40 to 49 years and between groups of ≤ 39 years and 50 to 59 years. However, K was significantly different only in comparison between age groups of ≤ 39 years and 60 to 69 years, groups of ≤ 39 years and 70 to 79 years and groups of ≤ 39 years and 80 to 89 years. Significant differences were also found in age, ACD and K between each AL group (F = 34.30, 58.74 and 66.79 and for age, ACD and K, respectively; *P* < 0.001 for all parameters) (Table 2). Post hoc pair-wise comparisons showed that age was significantly different in every comparison between AL groups. ACD was significantly different in every comparison except between AL groups of 25 to 25.99 mm and > 26 mm. K was significantly different in every comparison except between AL groups of 24 to 24.99 mm, 25 to 25.99 mm and AL > 26 mm.

**Table 1:** Comparisons of axial length (AL), anterior chamber depth (ACD), keratometry (K) between various age groups

Age (yr)	20–39	40–49	50–59	60–69	70–79	≥80	F	*P* value^a^
	(*n* = 22)	(*n* = 40)	(*n* = 123)	(*n* = 210)	(*n* = 301)	(*n* = 104)		
AL (mm)	24.83 ± 1.00	24.52 ± 1.06	23.90 ± 0.85	23.59 ± 0.90	23.38 ± 0.75	23.24 ± 0.64	31.84	<0.001
ACD (mm)	3.57 ± 0.67	3.60 ± 0.39	3.27 ± 0.43	3.06 ± 0.42	2.87 ± 0.41	2.74 ± 0.36	45.01	<0.001
K (diopters)	43.14 ± 1.45	43.85 ± 1.40	43.93 ± 1.39	44.16 ± 1.31	44.30 ± 1.40	44.39 ± 1.43	4.58	<0.001

Values are presented as mean ± SD or number unless otherwise indicated

^a^One-way ANOVA

**Table 2:** Comparisons of age, anterior chamber depth (ACD), keratometry (K) between various axial length (AL) groups

AL (mm)	<23	23–23.99	24–24.99	25–25.99	≥26	F	*P* value^a^
	(*n* = 212)	(*n* = 358)	(*n* = 175)	(*n* = 37)	(*n* = 18)		
Age (yr)	71.29 ± 8.95	68.99 ± 10.71	63.39 ± 12.36	55.43 ± 17.88	51.00 ± 13.23	34.30	<0.001
ACD (mm)	2.73 ± 0.41	3.00 ± 0.40	3.24 ± 0.40	3.52 ± 0.53	3.62 ± 0.63	58.74	<0.001
K (diopters)	45.14 ± 1.23	44.18 ± 1.15	43.31 ± 1.25	43.09 ± 1.42	43.00 ± 1.40	66.79	<0.001

Values are presented as mean ± SD or number unless otherwise indicated

*AC* anterior chamber, *D* diopter

^a^One-way ANOVA

Multivariate analysis revealed that advancing age has significant association with shorter AL (*P* < 0.001) and ACD (*P* < 0.001), although the association with K was not significant (*P* = 0.398). The inverse correlation between AL and K was also significant in multivariate analysis (*P* < 0.001).

## Discussion

In the present study, univariate analysis and ANOVA showed increased age had inverse correlation with AL and ACD, and had positive correlation with K, although the correlation between age and K was insignificant in multivariate analysis. AL had a negative correlation with K that was significant both in univariate and multivariate analyses.

Previously, studies have been performed to investigate the relationship between age and ocular biometry including AL, ACD and K. However, there exist controversies in the results between the studies. As in the present study, several studies have reported age-related decreases in AL [6, 12, 16–19]. However, other studies have reported no age-related decrease in AL [8–10], although two of the studies revealed age-related decrease in ACD [9, 10].

In a study including a large population of Chinese adults, Wong et al. [6] reported that older subjects had shorter AL, shallower ACD, shorter vitreous chamber depth (VCD), but thicker lens and more severe cataract. A survey of 220 adult Chinese showed a decreasing trend of AL and VCD with advancing age [19], and shorter ALs in older compared with younger subjects were also observed among Eskimos in Alaska [18]. A decrease in mean AL with increasing age was also detected in participants of Blue Mountain Eye Study who were representative of the Australian population [17]. By contrast, studies including Mongolians and Latinos in the USA, respectively, showed no age-related difference in AL [8, 9].

The mechanism explaining shorter ALs in older persons is still unclear. It is unclear that shorter ALs in older persons is due to an actual reduction in AL with aging or is just caused by a cohort effect that older age group could have smaller eyes because of poorer nutrition and general health [6]. Although some studies suggested that AL remains stationary since adolescents [10, 20], other studies indicate that a reduction in AL with aging is possible [12, 16]. Grosvenor et al. [16] proposed that age-related reduction in AL occurs as an emmetropization mechanism, compensating for the increase in the refractive power caused by the change of the lens [16]. By contrast, Ooi et al. [10] reported that there was no significant age-related difference in AL or K, and suggested that age-related decrease in the gradient-index of the lens acts as an emmetropizing mechanism by compensating for the steepening of both the front and back lens surfaces. Due to the cross-sectional nature of the present study, whether the results are just due to cohort effects or actual changes in the ocular biometry cannot be determined. Therefore, further prospective studies with long-term follow up are warranted to investigate the chronological changes of ocular biometry and to distinguish between cohort effect and age-related changes.

ACD conceivably has close correlation with AL, because a larger eye may have deeper anterior chamber. In this study, multivariate analysis revealed that shallower ACD had significant association with older age even after controlling the effect of AL, which is consistent with the results of the previous studies [6, 10, 16, 19, 21]. This trend may be due to the thickening of the lens due to progression of cataract with advancing age, which was proven in previous studies [6, 10]. Due to the increase in lens thickness, VCD was also shorter in older persons [6, 10, 19]. Although all patients in this study did have cataract, the difference in the severity and nature of cataract, i.e., younger patients tend to have anterior or posterior subcapsular cataract that does not induce significant lens thickening, whereas older patients tend to have nuclear sclerosis that lead to progressive lens thickening, might have influence on the results.

Multivariate analysis revealed that the *K* value was not significantly associated with age, which is in good agreement with the results of other studies that corneal curvature remains stable with age [6, 8, 10, 22]. Meanwhile, the results showed negative correlation between AL and K both in multivariate and univariate analyses. The inverse relationship between the two parameters may be due to emmetropization mechanisms during the childhood, as normal eye development involves matching between components, and flattening of cornea occurs to compensate for AL elongation during eye growth [23]. These findings indicate that cornea curvature changes in harmony with AL growth in childhood, and remain unchanged with advancing age, supporting the postulation that there is no progressive shortening of AL with aging, since this can lead to a substantial hyperopic shift without change in corneal curvature, which is not a universal feature in old age. Studies revealed that hyperopic change begins at around 40 years, and myopic shift occurs at around 70 years [13–15]. Otherwise, change in the configuration and thickness of the lens with aging may play role in age-related changes of refractive error, which could compensate for the possible AL change. We believe further prospective studies can determine the exact mechanism of changes in ocular biometry related to aging.

The present study has limitations as follows: 1) This study excluded refractive error in the analyses, although it is closely associated with *AL* and *K* value. However, as this study included patients with various severity of cataract, which is a confounding factor in the evaluation of the relation between refractive error, *AL* and *K* value. Moreover, emmetropization mechanism that occurs even in adults can attenuate the effect of changes in ocular parameters on refractive error [23]. Fotebar et al. [17] also reported that differences in AL were not significantly associated with changes in refractive error. We also excluded patients with high degree of myopia or hyperopia as well as those with extremely short or long AL to eliminate the effect of pathologic AL changes. 2) We did not evaluate the effect of height, although prior studies showed that height had significant association with AL and K [7, 13, 24]. Taller stature was significantly associated with longer AL, deeper ACD and flatter cornea in studies including a large population of Caucasians and Asians [7, 13]. Considering that older people tend to have short stature compared to younger ones, inclusion of height in the analyses may be helpful for the determination of the cohort effect. For instance, although a decreasing trend in AL with increasing age was found in a former study, the difference became insignificant after adjustment for height [13]. 3) We excluded the cases with accompanying ocular pathologies that can affect the AL or ACD when the history of the ocular pathologies was described in medical records. However, due to the retrospective nature of the present study, there is possibility that cases with other ocular pathologies were included and caused bias when the pathologic conditions were not recorded in the patient chart, especially in patients younger than 50 years of age who were more likely to have other ocular pathologies associated with cataract. 4) In the present study, there was no influence of gender on ocular biometry. However, several studies reported that gender has significant association with ocular biometry [6, 9, 13]. 5) As this study included only patients with cataract, the results have limited value to be applied in general population. Therefore, we believe further prospective larger population-based studies that include normal subjects are needed to address these limitations.

## Conclusion

This study showed that older age had association with shorter AL and shallower ACD, and AL had an inverse correlation with K. Further studies are needed for the elucidation of these phenomena.

## Abbreviations

ACD: Anterior chamber depth
AL: Axial length
ANOVA: One-way analysis of variance
K: Keratometry
RPE: Retinal pigment epithelium
VCD: Vitreous chamber depth
